# Supplementary material for: HIVprotI: an integrated web based platform for prediction and design of HIV proteins inhibitors
Source: J Cheminform. 2018 Mar 9;10:12. doi: 10.1186/s13321-018-0266-y (PMC5845081; doi:10.1186/s13321-018-0266-y)
Supplement: Supplementary file 2 — Additional file 2. Source code of HIVProtI web server. [file 13321_2018_266_MOESM2_ESM.zip › HIVprotI_Source-code/hivprotI/weka-3-6-6/doc/weka/core/Undoable.html]

Undoable


---


|  |  |  |
| --- | --- | --- |
| **Overview** \| **Package** \| **Class** \| **Tree** \| **Deprecated** \| **Index** \| **Help** \| **Weka's home** | |  |
| **PREV CLASS**   **NEXT CLASS** | **FRAMES**    **NO FRAMES**     **All Classes** |  |
| SUMMARY: NESTED \| FIELD \| CONSTR \| METHOD | DETAIL: FIELD \| CONSTR \| METHOD |  |


---


## weka.core Interface Undoable

**All Known Implementing Classes:**: ArffPanel, ArffSortedTableModel, ArffTableModel

---

``` public interface Undoable ```

Interface implemented by classes that support undo.

**Version:**
:   $Revision: 1.2 $

**Author:**
:   FracPete (fracpete at waikato dot ac dot nz)

---

| **Method Summary** | |
| --- | --- |
| `void` | `addUndoPoint()`             adds an undo point to the undo history |
| `boolean` | `canUndo()`             returns whether an undo is possible, i.e. |
| `void` | `clearUndo()`             removes the undo history |
| `boolean` | `isUndoEnabled()`             returns whether undo support is enabled |
| `void` | `setUndoEnabled(boolean enabled)`             sets whether undo support is enabled |
| `void` | `undo()`             undoes the last action |

**Method Detail**

### isUndoEnabled

```
boolean isUndoEnabled()
```

:   returns whether undo support is enabled

---


### setUndoEnabled

```
void setUndoEnabled(boolean enabled)
```

:   sets whether undo support is enabled

---


### clearUndo

```
void clearUndo()
```

:   removes the undo history

---


### canUndo

```
boolean canUndo()
```

:   returns whether an undo is possible, i.e. whether there are any undo points
    saved so far

    :   **Returns:**: returns TRUE if there is an undo possible

---


### undo

```
void undo()
```

:   undoes the last action

---


### addUndoPoint

```
void addUndoPoint()
```

:   adds an undo point to the undo history


---


|  |  |  |
| --- | --- | --- |
| **Overview** \| **Package** \| **Class** \| **Tree** \| **Deprecated** \| **Index** \| **Help** \| **Weka's home** | |  |
| **PREV CLASS**   **NEXT CLASS** | **FRAMES**    **NO FRAMES**     **All Classes** |  |
| SUMMARY: NESTED \| FIELD \| CONSTR \| METHOD | DETAIL: FIELD \| CONSTR \| METHOD |  |


---
